# Global circumferential left ventricular strain impairment in hypertrophic cardiomyopathy: comparison to left ventricular hypertrophy and late gadolinium enhancement

**DOI:** 10.1186/1532-429X-15-S1-E122

**Published:** 2013-01-30

**Authors:** Laurent Macron, Alban Redheuil, Golmehr Ashrafpoor, Nadjia Kachenoura, Emilie Bollache, Albert A Hagège, Michel Desnos, Pierre Croisille, Patrick Clarysse, Elie Mousseaux

**Affiliations:** 1 Radiology, Cardiovascular Imaging Unit, HEGP,APHP, Paris, France; 2 Cardiology, HEGP,APHP, Paris, France; 3 LIF, INSERM U678, UPMC, Paris, France; 4 CREATIS, UMR CNRS 5220 - INSERM U1044, Lyon, France

## Background

to evaluate the relationship between Left Ventricular (LV) myocardial strain, mass, wall thickness and the extent of fibrosis in hypertrophic cardiomyopathy (HCM)

## Methods

Forty HCM patients and 20 matched controls underwent a comprehensive CMR including cine imaging, late gadolinium enhancement (LGE) and short axis tagging. Global peak circumferential LV strain (Ecc) was generated from tagging sequences using InTag®. LGE volume was quantified semi automatically using a 6SD threshold.

## Results

HCM patients (50±18 years, 65% men) had normal LVEDV (149±46mL, p=0.24), LVESV (52±24mL, p=0.78) and LVEF (65±11%, p=0.38). LV mass (198±69g, p<0.001) and LV mass index (108±37g/m2 p=0.002) were significantly increased, resulting in decreased LV mass/LV volume ratio (1.40±0.54, p=0.005) in HCM compared to controls. Median maximal wall thickness was 19.6 (14.4 to 32.3mm). In HCM, LGE was present in 32/40 (80%) and mean LGE mass was 4.31±4.94g.

Ecc was significantly impaired in HCM patients (-8.82±0.32 vs. -15.54±2.54%, p<0.0001)

Ecc impairment was significantly associated with increased LV mass index (r=0.51, p=0.0009), LV mass/ LV volume ratio (r=0.67,p<0.0001) and LV maximal wall thickness (r=0.51, p=0.008). Moreover, Ecc impairment was associated with increased LGE mass (r=0.39, p=0.01).

## Conclusions

Global LV circumferential myocardial deformation was strongly decreased in HCM and significantly associated with LV hypertrophy and the extent of LGE.

## Funding

none

